# Asynchrony of ageing among traits in a wild bird population

**DOI:** 10.1093/evlett/qrag029

**Published:** 2026-07-21

**Authors:** Claire Lok Sze Tsui, Michael Briga, Jan Komdeur, Terry Burke, David S Richardson, Hannah L Dugdale

**Affiliations:** School of Biological Sciences, University of East Anglia, Norwich, United Kingdom; Behavioural and Physiological Ecology, Groningen Institute for Evolutionary Life Sciences, University of Groningen, Groningen, Netherlands; Department of Biology, University of Turku, Turku, Finland; Turku Collegium for Science, Medicine and Technology, University of Turku, Turku, Finland; Behavioural and Physiological Ecology, Groningen Institute for Evolutionary Life Sciences, University of Groningen, Groningen, Netherlands; Department of Animal and Plant Sciences, University of Sheffield, Sheffield, United Kingdom; School of Biological Sciences, University of East Anglia, Norwich, United Kingdom; Nature Seychelles, Victoria, Mahé, Republic of Seychelles; Behavioural and Physiological Ecology, Groningen Institute for Evolutionary Life Sciences, University of Groningen, Groningen, Netherlands

**Keywords:** ageing, senescence, selection pressure, Seychelles warbler

## Abstract

Ageing i.e., age-related changes in a trait, is a highly variable process. Studies have investigated variation in ageing among species and individuals, but little is yet understood about variation between traits. Evolutionary hypotheses argued that traits should age synchronously as selection should improve the trait that first senesces, therefore leading to trait synchrony. However, some past studies have demonstrated that traits do not senesce synchronously. We tested for the (a)synchrony of ageing across 13 reproductive, behavioral, physiological, and morphological traits in a population of wild Seychelles warblers (*Acrocephalus sechellensis*). We modeled ageing trajectories for each trait and quantified synchrony between traits with similar ageing trajectory shapes by comparing the onset and rate of ageing and running bivariate models to quantify covariation between traits. We found that five traits exhibited ageing while eight did not. Upon visual comparison of ageing trajectories, there were three groups of traits that shared ageing trajectories of the same shape. There was no support for synchronous ageing among any traits. Our study adds to growing evidence that ageing is asynchronous, and that the theory of synchronous ageing was erroneous. Our finding of senescence in direct fitness indices, such as survival and reproductive success but not in wing length or tarsus length gives weak support to newer evolutionary theories of asynchronous ageing stating that traits more strongly associated with fitness age faster.

## Introduction

Ageing is the age-related change of traits, while senescence is the deterioration of function and fitness associated with advancing age due to the accumulation of molecular, physiological, and cellular damage (Kirkwood & Austad, 2000; Monaghan et al., 2008). Ageing is widespread and highly variable across taxa, among populations, and between individuals (Cambreling et al., 2025; Jones et al., 2014; Nussey et al., 2013; Sanghvi et al., 2024). Even within individuals, traits can differ in their ageing trajectories—the age of onset and rate of senescence (Hayward et al., 2015). Differing within-individual ageing trajectories between traits is known as asynchronous or mosaic ageing (Walker & Herndon, 2010). The most notable example of asynchronous ageing is menopause (Ricklefs & Finch, 1995; Walker & Herndon, 2010). To date, there is still limited knowledge explaining the evolution of asynchrony of ageing. As age-related declines in traits may vary in their contribution to actuarial senescence, further knowledge of patterns of differential ageing across traits is needed to fully understand how ageing rates across various traits underlie or contribute to actuarial senescence. Through studying asynchronous ageing, we can better understand how variation in ageing affects age structures in a population. This is, in turn, important as a population’s age structure hugely influences a population’s demography, which drives evolution (Charlesworth, 1994; Jones et al., 2014).

Previous literature proposed that traits within an individual should senesce synchronously i.e., with the same age of senescence onset (Maynard Smith, 1962; Williams, 1957). This was based on the idea that senescence was caused by generalized deterioration, not from major changes in any one system (Williams, 1957). Selection should act to improve (delay onset) the first senescing trait that impairs fitness and function, thus synchronizing the onset and rate of senescence (Maynard Smith, 1962; Moorad & Ravindran, 2022; Williams, 1957). More recently, Hayward et al., (2015) interpreted the theory as there being a critical threshold for each trait value, which if crossed, will result in death. Thus, selection will be strongest on traits closest to the threshold value (Hayward et al., 2015). Gaillard & Lemaître (2017) suggested that selection pressures on traits should differ according to their early life survival, thus aligning ageing trajectories. Regardless of the importance of traits to survival and reproduction, these interpretations of the original idea indicate that selection does not act on all traits uniformly, but rather be trait-specific (Gaillard & Lemaître, 2017).

There is considerable empirical work providing evidence for the asynchrony of ageing across traits (Moullec et al., 2023), but, to the best of our knowledge, none supporting Williams’ theory of synchronous senescence. Health and lifespan are commonly found uncoupled in humans and laboratory model organisms (Bansal et al., 2015; Christensen et al., 2009). Within individuals, traits differ in onset and rate of ageing in various taxa under both wild and captive conditions, such as plants such as plants, lab organisms, mammals, birds, and snakes (Boonekamp et al., 2018; Briga & Verhulst, 2021; Cayuela et al., 2020; Cooper et al., 2021; Hayward et al., 2015; Herndon et al., 2002; Moullec et al., 2023; Rodríguez et al., 2017). Most studies showed asynchrony of senescence in reproductive and survival traits (e.g., Bouwhuis et al., 2009; Cayuela et al., 2020; Cooper et al., 2021; Fay et al., 2021; Naciri et al., 2022), while two found asynchrony in physiological traits (Boonekamp et al., 2018; Briga & Verhulst, 2021). Additionally, Hayward et al. (2015) studied whether traits within a functional grouping (e.g., reproductive, survival, and behavioral) senesced more similarly in Soay sheep (*Ovis aries*), but did not detect any synchrony of ageing. However, the lack of empirical studies supporting the synchrony of ageing calls for an update to the old verbal theory.

More recent theories on (a)synchrony of ageing have hypothesized that traits age faster, where a decline in trait function causes greater fitness (here defined as lifetime reproductive success weighed by age-specific contribution to population growth) loss (Boonekamp et al., 2018; Briga & Verhulst, 2021). Traits with the greatest marginal contribution to fitness should show the least marginal change with age, since fitness costs from these traits senescing will be the highest (Rodríguez‐Muñoz et al., 2019). Similarly, weaker selection on traits less important to fitness components would lead to comparatively earlier ageing in those traits, since optimal resource allocation would delegate less resources to repair that trait, leading to asynchronous ageing (Rodríguez‐Muñoz et al., 2019). Counterintuitively, a theoretical study found that traits under the strongest selection in early life have the fastest rate of decline of age-specific selection, which leads to faster ageing rates for these traits (Moorad & Ravindran, 2022). Trade-offs and resource allocation are also hypothesized to explain asynchronous ageing (Cohen et al., 2020). Traits or tissues receiving more energy for repair would senesce slower, or somatic tissues would senesce faster when more energy is allocated towards reproduction (Romero-Haro et al., 2025).

While the difference in selection pressure has been the most reviewed cause, genetic correlations among traits between and within age classes may also impact asynchrony of ageing, depending on the selection pressures on respective traits (Hayward et al., 2015; Moorad & Ravindran, 2022). Negative genetic correlations between traits in late life may lead to accelerated ageing rates for one trait and slower ageing rates for the other trait, while positive pleiotropy between traits may synchronize their ageing rates (Charlesworth, 2001; Hayward et al., 2015; Williams, 1957). Nonevolutionary factors may also contribute to apparent asynchrony of senescence. Traits may vary in the extent of damage they accumulate from environmental factors over time, causing differing rates of senescence (Moorad & Ravindran, 2022). However, only a few studies have investigated the synchrony of senescence across a broad range of traits. Here, we address this gap by testing asynchrony of senescence across a wide range of traits in a longitudinal wild bird population.

The Seychelles warbler (*Acrocephalus sechellensis*) (hereafter SW) population on Cousin Island provides an exceptional longitudinal study system. Owing to bi-annual sampling for c.a. 30 years, the lack of (im)migration (<0.1%) (Komdeur et al., 2004), and high mean resighting probability (Brouwer et al., 2010), we have comprehensive life-history data for individuals from birth to death, accurate survival data, and longitudinal measures of numerous traits (Sparks et al., 2022). Importantly, these repeated individual measures allow us to account for selective disappearance by statistically separating within- and between-individual trait variation (van de Pol & Verhulst, 2006). Given the lack of predators of adults on the island, there are low levels of adult extrinsic mortality contributing to annual mortality (van de Crommenacker et al., 2017). Consequently, the SW is relatively long-lived with a mean lifespan of ca. 4.4 years post fledgling (Figures S1 and S2), and a maximum of 19 years (Hammers & Brouwer, 2017).

The warblers exhibit actuarial, reproductive (annual reproductive success) and telomere length senescence (Barrett et al., 2013; Hammers et al., 2013, 2015), and ageing in provisioning rate (Hammers et al., 2021). Reproductive senescence begins at 6 years for females without helpers (Hammers et al., 2012) and 7.8 years for males (Raj Pant et al., 2020), respectively. These features make this population an excellent study system for investigating (a)synchronous ageing. In this study, we aim to (1) quantify the ageing trajectories of 13 traits in the SW including morphological, physiological behavioral, and fitness-related traits and (2) test for their (a)synchrony of ageing. We expect traits to age asynchronously, sharing a low degree of synchrony and ageing trajectories to be dissimilar between traits. We also expect a lack of overall pattern across the functional groupings of traits. This study will help us identify wider patterns in variation in ageing between traits, and enable us to further understand the drivers of evolution of asynchronous ageing.

## Methods

### Study system

SWs are small (13–14 cm) passerine birds endemic to the Seychelles. On Cousin island (4.3315°S, 55.6620°E) the population of this facultatively cooperative breeder is structured into ca. 115 territories each containing 1 breed group that consists of a dominant breeding pair, offspring, and may include helper or nonhelper subordinates (Richardson et al., 2002). The Cousin population of c.a. 320 individuals has been monitored more intensively since 1985 (Komdeur, 1992; Komdeur & Daan, 2005). Since 1997, ~ 96% of the individuals have been ringed with a unique British Trust for Ornithology (BTO) ring and color ring combination (Richardson et al., 2001; Sparks et al., 2022).

Fieldwork occurs twice a year, in the minor (January–March) and major (June–October) breeding seasons. Individuals were captured using mistnets and playback. Unringed birds were ringed with a BTO metal ring and a unique color ring combination. Ca 70 µl of blood was collected via brachial venipuncture (Brown et al., 2021b). These blood samples have previously been used for molecular sexing and genetic parentage assignment (Richardson et al., 2001; Sparks et al., 2022), measuring malaria presence (Hammers et al., 2016), buffy coat (Brouwer, 2007), telomere length (Barrett et al., 2013; Brown et al., 2022), haematocrit (Brown et al., 2021b), oxidative stress, and antioxidant capacity (van de Crommenacker et al., 2011). Unringed individuals were aged using a combination of hatch date, behavior, observations in the nest, and eye color as confirmation if hatch date was unknown (Komdeur, Bullock & Rands, 1991) . Morphological traits were measured including body mass (± 0.1 g), wing length (± 0.1 mm), right tarsus length (± 0.1 mm) and fat score (0–5).

Individual territories were visited every 2 weeks to carry out a population census. Survival and social status were determined by observing color-ringed birds and following the dominant female of each territory for 15 min (Crommenacker et al., 2011; van Boheemen et al., 2019). The dominant breeders were defined as the pair-bonded male and female in a territory according to interactions and nesting behavior, while subordinates were defined as helpers or nonhelpers depending on whether they displayed brooding or offspring provisioning (Richardson et al., 2002). If a nest was found in the territory, the nest was checked every week to determine nest stage (Komdeur & Daan, 2005). 60–90 min nest watches were carried out during the incubation and provisioning stage of nesting (van Boheemen et al., 2019). Provisioning rates of individuals were calculated as the total number of provisioning events per bird over the duration of the watch. Watches where more than 10% of provisions were by unidentified birds were excluded to ensure accuracy of the nest watch (van Boheemen et al., 2019).

Food (insect prey) abundance was measured according to Komdeur (1996), and mean insect availability was calculated from averaging insect abundance across the island in a year or season. Territory quality was defined as the insect availability within a territory (Hammers et al., 2015), and was calculated as α·∑(c_x_i_x_), where α is the territory size (hectares), c_x_ is the foliage cover for broad-leafed tree species x, and i_x_ is the mean monthly insect count for tree species x per unit leaf area dm^2^ (Komdeur, 1992, 1996).

### Measurement of physiological and fitness traits

Oxidative stress (ROMs) and antioxidant capacity (OXYs): undertaken as part of a previous study (Brown et al., 2021b; van de Crommenacker et al., 2011). Plasma from each blood sample was separated by centrifugation (9,889 × *g* for 8 min) and frozen. The oxidative damage and antioxidant compounds were quantified using d-ROMs test kit (Diacron, Grosseto, Italy) and OXY-Adsorbent test kits (Diacron) respectively (van de Crommenacker et al., 2011). Full assay details can be found in van de Crommenacker et al. (2011b). ROMs were calculated as the sum of oxidative damage compounds while OXYs were calculated as the sum of antioxidant compounds.

Haematocrit and Buffy coat: Within ca. 3 hr of bleeding, the heparinized capillary tube was centrifuged for 8 min at 6,000 × *g* (Brown et al., 2021b). Haematocrit was measured as the proportion of erythrocytes to the whole blood volume in the capillary tube with calipers to the nearest 0.01 mm. Buffy coat was measured under a 10x magnifying glass as the proportion of leukocytes and platelets to the whole blood volume.

Malaria: Blood samples were tested for the presence or absence of haemosporidian parasite infection using a nested polymerase chain reaction (PCR) technique (Hammers et al., 2016; Hellgren et al., 2004) run twice per sample. Individuals with conflicting test results from the test consensus were removed from the dataset. 87% of samples had consistent test results across both PCR runs (Fairfield et al., 2016).

Telomere length: We used the telomere dataset from Spurgin et al. (2018) for samples collected between 1995 and 2014. A quantitative polymerase chain reaction (qPCR) method was used to estimate relative telomere length (RTL; Barrett et al., 2013). Within and between plate repeatability for all samples screened over different years was 0.74 (95% CI = 0.74–0.75) and 0.68 (95% CI = 0.65–0.70), respectively (Sparks et al., 2022). There were no detected blood sample storage time effects on telomere length (Spurgin et al., 2018).

Annual reproductive success (ARS): Blood samples were genotyped and parentage was assigned using package *MasterBayes 2.52*, from which a pedigree was constructed (Hadfield et al., 2006). Details of the parentage analysis and pedigree construction can be found in Sparks et al. (2022). ARS was quantified as the number of offspring in the genetic pedigree assigned to a parent with ≥ 80% genetic confidence in a year (Sparks et al., 2022).

Survival: Given the high resighting probabilities in this population (of 0.92 ± 0.02 for individuals ≤ 2 years and 0.98 ± 0.01 for older individuals) (Brouwer et al., 2010), birds not seen for two consecutive field seasons were assumed dead unless resighted thereafter.

### Statistical analysis

Statistical analyses were conducted in R 4.2.2 (R Core Team, 2022). We analysed ageing trajectories of all 13 traits (Table 1) with data collected between the years 1981 and 2022, using mixed Generalized additive models (GAMs) using the package *mgcv* 1.9 (Wood, 2017). The sample sizes and distributions according to lifespan can be found in Table S1 and  Figure S4. GAMs allowed us to fit nonparametric smoothing terms between a trait and age, which were more suitable as it allowed for combined testing of gradual and threshold changes with age due to its flexible nature, as observed e.g., in body mass and metabolism (Briga et al., 2019; Douhard et al., 2017; Nussey et al., 2011, Briga & Verhulst, 2021 ). All models were fitted with a smoothing term for age (main effect) using a penalized thin plate regression spline, and the smoothing parameter was selected using restricted maximum likelihood. We initially fitted the maximum k to allow for flexibility of the model, and subsequently used gam.check() to test the fit of different values of k to select the best value. Selective appearance and/or disappearance can obscure apparent senescence patterns if the relationship between trait values in older age and lifespan are stronger in certain traits, and the within- and between-individual differences are not distinguished (van de Pol & Verhulst, 2006). We controlled for selective disappearance by fitting lifespan as fixed effect and bird identity as random effect for all traits except for ARS (van de Pol & Verhulst, 2006). Age of first and last reproduction (AFR and ALR, respectively) but not lifespan was fitted in the ARS trait model to account for selective disappearance. Lifespan was not fitted in the GAM for survival as individuals only die once. The selective disappearance terms (lifespan, AFR, and ALR) were initially fitted as smooth terms, but refit as linear terms where the term was linear (estimated degrees of freedom = 1). Additionally, we tested for age-dependent selective disappearance of individuals by including an age-by-lifespan interaction (Tables S27–S38), excluding survival due to age and lifespan being completely colinear in the trait. Four traits showed a significant age by lifespan interaction, namely body mass, malaria, provisioning rate, and ARS (Tables S27, S35, S37, S38). However, due to incompatibility with the downstream analyses, we could not include the age-by-lifespan interaction models in the main results.

**Table 1:** Rationale for phenotypic trait inclusion and predictions of their ageing trajectory according to the findings of Moorad & Ravindran (2022).

Trait	Rationale for inclusion	Prediction (direction and degree)
Body mass	Body mass is the most simple and common indicator of body condition (Labocha & Hayes, 2012). Many studies attributed body mass ageing to senescence in function and condition (Nussey et al., 2011).	Declines more as body condition is directly important to fitness
Wing length	Indicator of flight ability and feather quality, may affect a bird’s foraging ability and thermoregulation (Kiat & Sapir, 2018).	Declines to a lesser degree as SW do not use flight much
Right tarsus	Treated as a control of ageing trajectory as tarsus length is developmentally determined and not known to senesce (Tjørve & Tjørve, 2010).	No change
Fat score	Fat score indicates energy reserves and condition in birds (Labocha & Hayes, 2012).	Slight decline similar to body mass as an indicator of body condition
Oxidative stress (ROMs)	Damages biological macromolecules, which may cause accelerated ageing and degenerative diseases (Beckman & Ames, 1999; Finkel & Holbrook, 2000).	Increases to a similar slope as OXYs declines.
Antioxidant capacity (OXYs)	Buffers against oxidative stress, which may help individuals avoid the harmful consequences of oxidative stress (Felton & Summers, 1995).	Declines similarly to ROMs increase
Haematocrit	Reflects haemoglobin concentration. An indicator of aerobic capacity, which is important in endurance and performance in SW (Brown et al., 2021b).	Declines similarly to oxidative stress
Buffy coat	Refers to the white blood cell layer after centrifugation. Measure of leucocyte content in blood, which may be indicator of immune function and survival (Møller & Saino, 2004; Mondragão-Rodrigues & Macedo, 2023).	Declines at the same time as malaria prevalence increases due to both traits being immune indicators
Malaria presence	Potential indicator of immune response against infection in SW (Hammers et al., 2016).	Decreases then increases at a similar age to buffy coat decrease
Telomere length	Biomarker of senescence that declines with age and predicts survival in SW (Barrett et al., 2013)	Declines similarly to survival
Provisioning rate	Declines in female, but not male SWs, with age and offspring first year survival declines with parent age (Hammers et al., 2021)	Increases with experience then decreases, but less than ARS
Annual reproductive success (ARS)	Senesces in SW (Hammers et al., 2012; Raj Pant et al., 2020).	Increases with experience then declines. Senesces more as it is a direct fitness index
Annual survival	Senesces in the SW (Hammers et al., 2013).	Declines after age 6. Also senesces more as it is a direct fitness index

We fitted annual insect abundance, social status (dominant or subordinate), and sex as control variables for all models. Food abundance is an indicator of available resources on the island and energy acquisition in the warblers (Hammers et al., 2012), and social status may affect a bird’s energy allocation and usage. Individual differences in the availability and allocation of these resources may influence senescence trajectories (Kirkwood, 1977). Sex was included as a fixed effect to account for possible sex differences in ageing (Brouwer et al., 2006). We expected the above control variables to impact ageing in a similar way, as we expect better environmental conditions or lower energy expenditure to correlate with lower ageing rates and vice versa. However, trait specific predictions may differ, as lower ageing rates in body condition would manifest as a lower decrease in body mass, while lower ageing rates in oxidative stress may present as a slower increase or a plateau in oxidative stress.

To test for an age by sex interaction, we fitted three models per trait as outlined in Pedersen et al. (2019)—model G, model GS, and model S. The model fit for each trait was compared using both Akaike’s Information Criterion (AIC) and the biological significance of the interaction to determine which model was most suitable. The model with the lowest AIC was considered as the best fit (ΔAIC < 4) (Burnham et al., 2011). Tarsus length was fitted in the body mass and wing length trait models to control for body size. Birth cohort was fitted as a random effect in all models. For all morphological traits, haematocrit, buffy coat, and provisioning rate models, observer identity was fitted as a random effect. Technician identity was fitted as a random effect for RTL. All response variables with a Gaussian distribution were z-transformed.

All models were checked for convergence and tested for concurvity with the *mgcv* package. The full results of the ageing models, including all fixed and random effects for each trait can be found in Tables S2–S14.

As GAMs do not produce interpretable or meaningful effect sizes or *p*-values for smooth terms, we calculated the first order derivatives of the age smooth term to determine which traits demonstrated ageing. Positive derivative values indicate a positive slope, usually suggesting improvements with age, vice versa for negative derivative values, while a value of 0 indicates maxima or minima. Traits reach maximum values when their first-order derivative is zero. Hence, we defined a trait’s ageing as statistically significant when the upper 95% confidence of the derivative crossed zero.

There are currently three main approaches to quantifying asynchrony of ageing. Hayward et al’s (2015) quantitative method works well to quantify features of ageing trajectories. However, by normalizing age-specific means, this approach may miss variation in ageing rates. Cooper et al’s (2021) quantitative approach also identifies whether ageing onset and ageing rates are determined by biologically meaningful trait values. However, this method may find asynchrony of senescence in traits with differing age-specific variances that have the same age-specific means. Results may therefore differ according to the methodology used to detect asynchrony of senescence or compare ageing rates (Moorad & Ravindran, 2022). The two methods outlined above both make indirect comparisons of ageing trajectories, which do not estimate synchrony of ageing. Differently, Briga & Verhulst (2021) directly quantifies synchrony of ageing by estimating within-individual correlation of ageing rates between traits.

We tested for asynchrony of ageing according to the methods outlined in Briga & Verhulst (2021). Trait ageing trajectories were compared first. We considered the ageing traits to have asynchronous ageing if traits showed differences in a combination of whether they showed significant age-related declines (as determined using derivatives), the number of knots (curviness of the spline > 2), age of onset in ageing (>2 years), slope direction, and effect size. If ageing traits showed similar ageing trajectory shapes and a similar age of onset (including different ageing rates or slopes), a bivariate model was run between the traits to estimate the covariance between the two traits to compare within individual ageing rates (Briga & Verhulst, 2021).

Initial comparison produced three groups of traits with similar ageing trajectories. We restricted the bivariate models to traits with age-related declines. We ran a bivariate model between body mass and provisioning rate using the package *mcgv*. While survival also shared a similar ageing trajectory, bivariate models were not run between survival and the other two traits as comparing a within-individual ageing trajectory (body mass and provisioning rate) and a between-individual ageing trajectory (survival) is not biologically meaningful. Across other traits, the pairwise correlations between the trait values are low (Figure S3).

## Results

### Ageing trajectories

Overall, We observed three broad ageing trajectory shapes. Body mass, wing length, oxidative stress, haematocrit in males, provisioning, and survival showed a quadratic relationship (Figures 1 and 2a, b, e, g, k, and m). Tarsus length, fat score, antioxidant capacity, and RTL and had a linear relationship (Figures 1 and 2c, d, f, and j). As expected, tarsus length is developmentally determined, thus was constant throughout life (Figures 1 and 2c). Haematocrit in females, buffy coat and malaria presence showed an initial decrease (until ages 3 and 5, respectively) and then levelled out (Figures 1 and 2g–i). Thus, there were no age-related declines as both traits remained steady with age after the initial decline. ARS did not share a similar ageing trajectory with other traits (Figure 1 and 2l).

**Figure 1:**
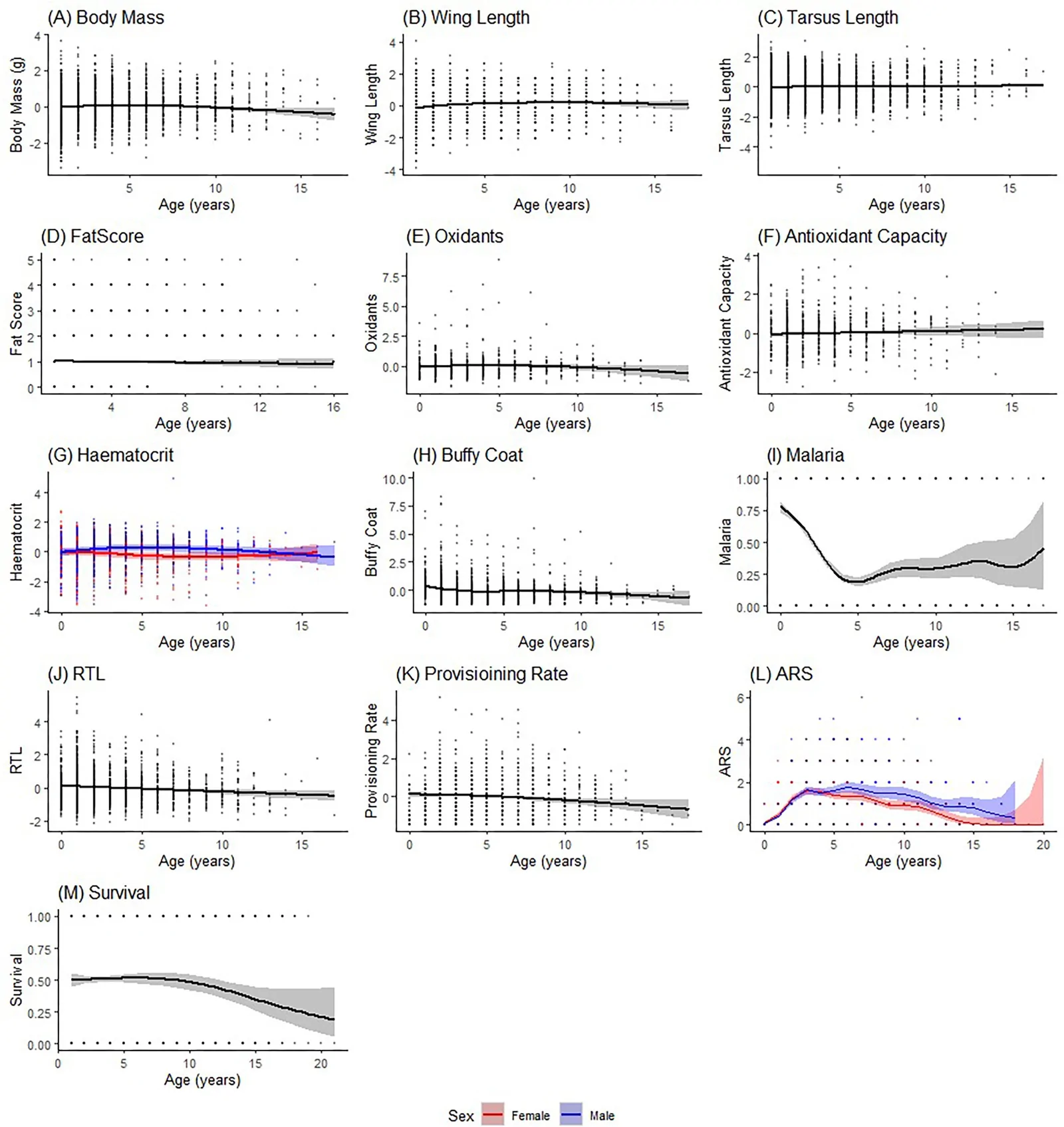
The age-specific trajectories of 13 phenotypic traits in individuals the Seychelles warbler population of Cousin Island (dates differ per dataset, minimum date 1981, maximum date 2022) identified through Generalized Additive Models. The *x*-axis is age in years, and the *y*-axis is the trait value; all traits, except for annual reproductive success, survival and malaria, are z-transformed. The solid black line depicts the model-predicted curve and the grey shaded areas depict the standard error. Black points are raw data points. RTL = relative telomere length. ARS = annual reproductive success. For figure 1g and 1l, the red curve corresponds to female ageing trajectory, while blue curve corresponds to male ageing trajectory.

**Figure 2:**
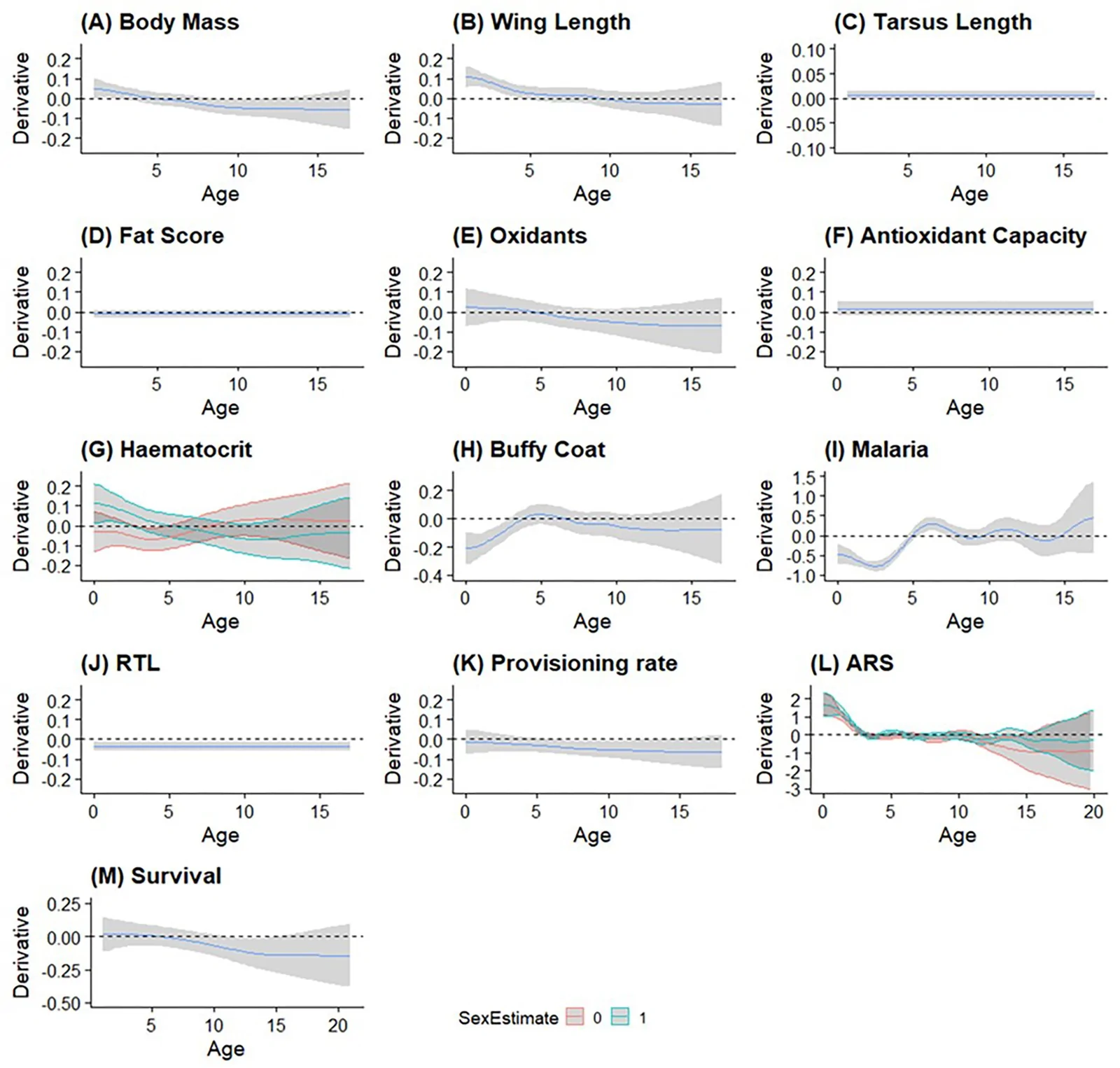
The ageing rates of the 13 phenotypic traits in individuals of the Seychelles warbler population of Cousin Island (dates differ per dataset, minimum date 1981, maximum date 2022). The rate of ageing of each trait was calculated by obtaining first-order derivatives (solid blue line) of the model-predicted curve of the age term in each Generalized Additive Model. On the *y*-axis is the derivative of the curve, with age (years) on the *x*-axis. The solid blue line depicts the ageing rate and the grey shaded areas around the blue line depict the 95% confidence intervals. A derivative value above 0 indicates the slope is positive, and vice versa. Traits with 95% upper confidence intervals that cross the dotted zero line demonstrate significant trait ageing. This includes (A) body mass, (J) RTL (relative telomere length), (K) provisioning rate, (L) ARS, and (M) survival. Blue trajectory in figure 2l indicates male ageing trajectory and red for females.

### Age-related declines

Five out of 13 traits, one of which was sex-specific, showed significant age-related declines: Body mass, telomere length, provisioning rate, ARS in females, and annual survival probability (Figure 1 and 2a, j, k, l, and m; Tables S2, S11–S14, respectively). Body mass and provisioning rate showed a quadratic relationship with age, initially increasing then decreasing postpeak from c.a. 8.5 and 6 years, respectively (Figures 1 and 2a and k; Table 2). Similarly, annual survival probability displayed an increase until the age of significant decline–11 years (Figures 1 and 2m; Table 2). Telomere length decreased linearly with age (Figures 1 and 2j, Table 2). ARS increased until age 3 then remains roughly constant from age 3 to 11, except displaying a small significant decrease at age 7, followed by a plateau, then a larger significant decrease after age 11 (Figures 1 and 2l; Table 2).

**Table 2:** Age of onset of decline for five phenotypic traits in the Seychelles warbler population on Cousin Island (dates differ per dataset, minimum date 1981, maximum date 2022) based on the derivatives of the curves obtained from the Generalized Additive Models for each trait with significant ageing trajectories (Figure 2a and j–m). The mean of the derivative of the slope indicates the estimated population mean age of ageing. The upper and lower 95% confidence intervals where the derivative of the slope crosses 0 estimates the variation around the mean age of ageing. For traits with the mean and lower CI values in the negative, we have designated the mean and lower age of onset of decline as 0. RTL = relative telomere length.

Trait mean and 95% CI	Body mass	RTL	Provisioning rate	Survival	ARS (in females)
Upper	8.5	0	6	11	7 and 11
Mean	4.7	0	0	6.2	6 and 10
Lower	1	0	0	0	2.8

There was little evidence for sex differences in ageing rate aside from ageing of ARS (Tables S16–S26, Figures S5 and S6). Of the traits that showed significant age-related changes, different age-specific trajectories were observed. (1) Body mass, provisioning rate, and annual survival had a quadratic trajectory (Figure 2a, k, and m). (2) RTL showed a linear trend (Figure 2j). (3) Buffy coat, and malaria prevalence decreased in early life and then stabilized in later life (Figure 2 h and i). ARS did not show a similar age-trajectory with any other traits (i.e., ages asynchronously to other traits; Figure 2l). The traits within these “similar trajectory” groups do not belong to the same functional grouping (e.g., morphological, physiological, and fitness traits) except for group 3 being immune related traits.

### Within individual covariance of senescence rate between traits

Among the traits showing age-related declines, only body mass, provisioning rate, and survival shared a similar ageing trajectory. Survival was not included as a response variable in any of the bivariate models as survival ageing is a between-individual estimate. The degree of synchrony of ageing between body mass and provisioning rate was low (−0.03178). The full model output can be found in Table S15.

## Discussion

We quantified ageing trajectories, and tested for asynchrony of ageing, across 13 phenotypic traits in the SW. We found age-related declines in five traits: body mass, RTL, provisioning rate, ARS, and annual survival probability. There were three groups of traits sharing similar ageing trajectories; (1) Quadratic with age: body mass, provisioning rate, and survival, (2) linear decline with age: telomere length, and (3) improves in early life and stabilizes in late life: haematocrit female, buffy coat, and malaria infection. However, the onset of ageing often differed between those traits that aged (Table 2), and the degree of synchrony of ageing was low between traits that shared similar trajectories. There was little evidence of sex differences in ageing apart from ARS (Figures 1 and 2). Among the groupings of traits that shared similar trajectories, there was no pattern of similarities in ageing in functional grouping (morphological, physiological, immune, behavioral, and fitness traits), aside from immune traits sharing a similar pattern. Overall, our study showed asynchrony of ageing in the SW.

### Phenotypic trait ageing in the SW

#### Morphological trait ageing (body mass, wing length, right tarsus length, and fat score)

Ageing of morphological traits has not been previously studied in the SW, but the present study revealed key patterns. First, the observed decline of both body mass (accounting for tarsus length) indicated body condition ageing in the SW. Most studies on body mass senescence in other species support our findings (Beirne et al., 2015; Douhard et al., 2017; Kroeger et al., 2018; Nussey et al., 2011), while a couple do not (Hämäläinen et al., 2014; Moullec et al., 2023). Similar to our results, the only study (to our knowledge) using fat score did not report ageing (Milenkaya et al., 2013). Wing length did not show significant ageing in SW, unlike previous findings in other species (Piliczewski et al., 2018; Moullec et al., 2023; Śliż, 2022). SW often prefer to hop or do short flights for foraging and do not disperse across islands (Komdeur et al., 2004), thus rely less on flight ability. This potentially results in negligible trade-offs between feather quality and other traits if the cost to maintain feather quality is lower than expected (Buttemer et al., 2020; Kiat & Sapir, 2018), explaining the lack of ageing. Overall, our findings suggested body condition ageing in the warblers, but not ageing in other morphological traits.

#### Physiological trait ageing (oxidative stress, antioxidant capacity, telomere length, and haematocrit)

Telomere length was the only physiological trait that showed ageing, which aligns with previous studies in the SW (Barrett et al., 2013; Brown et al., 2022), and many other species (Le Clercq et al., 2023; Remot et al., 2022). Additionally, telomere length senesces in the SW as short telomere length and higher telomere attrition rates predicted survival in the SW (Barrett et al., 2013; Brown et al., 2022).

The nonsignificant ageing of oxidative stress traits, i.e., ROMs and OXYs is consistent with past studies finding oxidative stress markers to be stable with age (Hindle et al., 2010; Hübner-Woźniak et al., 2011; Nussey et al., 2009), although another study found the opposite (Williams et al., 2008). As oxidative stress had the shortest timeframe of data collection (2006–2013), a lack of older individuals (age > 6, *N* = 114) may have led to a reduced power to detect ageing.

There was a significant decline with age in haematocrit in a previous study (Brown et al., 2021a). However, we did not find the same pattern in our study. This may be due to differences in modeling approaches. Studies in other species also found age-related declines in haematocrit content (Elliott et al., 2015; Goldberg et al., 2023; Hickmott et al., 2024). Since haematocrit does not predict survival in later life in the SW (Brown et al., 2021b), it likely does not senesce in this species. Overall, there was therefore little evidence of ageing in physiological traits in SW aside from RTL.

#### Immune trait ageing (malaria presence and buffy coat)

There was no evidence for age-related declines in immune traits in SW. Although previously thought to be attributed to selective disappearance (van Oers et al., 2010), the present study and Hammers et al. (2016) found little evidence for this. This suggests SW maintain immune function in late life (Hammers et al., 2015). The lack of ageing in buffy coat that our results showed further supports the lack of immune trait ageing in warblers, which differed to that found in the Soay sheep (Froy et al., 2019). More generally, due to studies failing to quantify within-individual change (Peters et al., 2019), the evidence for immunosenescence/ageing is mixed; with some finding ageing in immune traits (Cheynel et al., 2017; Peters et al., 2019) and others not (Bichet et al., 2022; Peters et al., 2019; Roast et al., 2022).

#### Behavioral trait ageing (provisioning rate)

The ageing trajectory of provisioning rate identified in the current study was similar to previous results in the SW, showing a decline in provisioning rate with age (Hammers et al., 2021). This aligns with results in another bird species (Krist et al., 2024), but contrasted with other studies (Cansse et al., 2024; Wilcoxen et al., 2010). In terms of implications for senescence of provisioning rate in the SW, although offspring first-year survival declines with breeder age in the SW (Hammers et al., 2021), it remains unclear whether this results specifically from age-related declines in provisioning rate, as breeder age may also affect offspring survival through factors like egg quality (Beamonte‐Barrientos et al., 2010).

#### Fitness-related trait ageing (ARS and survival)

Finding senescence in ARS and survival in the SW was expected based on previous results in SW (Hammers et al., 2015) and studies across a range of taxa (Cambreling et al., 2025; Jones et al., 2014; Lemaître & Gaillard, 2017; Tidière et al., 2018; Torres et al., 2011; Vágási et al., 2021; Vrtílek et al., 2022). The stability in ARS may partially be due to SW producing single-egg clutches in 91% of breeding attempts (Komdeur, 1996). Only 3% of SW had an ARS larger than two in our data, resulting in little variation in the cost of reproduction between breeders. Differences in estimated onset of reproductive senescence (age 11 here vs. 6 for females and 7.7 for males in earlier studies; Hammers et al., 2012; Raj Pant et al., 2020), likely stem from dataset differences and differences in modeling approach, as prior work excluded significant amounts of reproductive data gained in minor seasons and from subordinates (Hammers et al., 2012).

Survival probability ageing in our study shared the same trajectory shape with a previous SW study (Hammers et al., 2015), and is consistent with previous research across various taxa (Cooper et al., 2021; Gaillard & Lemaître, 2020; Hammers et al., 2015; Jones et al., 2014; Nussey et al., 2013). Unfortunately, survival senescence is a cross-sectional measure, since it is not possible to separate within and between individual heterogeneity in survival probability with a linear mixed model or GAM. To quantify survival senescence accurately, different approaches such as capture–mark–recapture models are required to account for individual variation in survival probability (Gimenez et al., 2018). Nonetheless, survival senescence is very well documented across taxa (Jones et al., 2014; Nussey et al., 2013). Overall, the ageing trajectories studied in the warbler largely aligned with past findings, demonstrating that the SW represents an excellent system to study ageing in the wild.

### Asynchrony of senescence

The asynchronous ageing of traits in the SW demonstrate that the original verbal theories on synchronous ageing by Williams (1957) and Maynard Smith (1962) are erroneous, and add to growing evidence of asynchrony of senescence in a wide range of taxa (e.g., Briga & Verhulst, 2021; Cayuela et al., 2020; Cooper et al., 2021; Hayward et al., 2015; Herndon et al., 2002; Tully, 2023; Tully et al., 2020). Apart from the similar ageing trajectory of immune related traits in male SW (Group 3), there is little evidence for functional grouping patterns in ageing across traits in the SW. This is consistent with the findings of Hayward et al. (2015) in the Soay sheep.

In terms of the evolutionary theory behind the asynchrony of senescence, this study provides weak evidence for the theoretical study that traits more associated with fitness undergo more rapid ageing (Briga & Verhulst, 2021; Moorad & Ravindran, 2022). We found age-related declines in direct fitness indices—i.e., survival and reproduction but not in traits less important to fitness, such as wing length, tarsus length, antioxidant, and oxidant capacity. However, the role of selection pressure as a driver of patterns of senescence in traits less consequential to fitness still remains unknown. Considering the ageing trajectory of body mass and immune traits (e.g., malaria presence) as examples, ageing was significant in the first trait but not in the latter. Two predictions can arise according to the newer theories suggesting trait-specific selection pressure explains ageing trajectories: (1) body mass ages faster because body mass is under stronger selection than immune traits and (2) immune traits do not age because they are under stronger selection than body mass. We can envision prediction one to be plausible; body mass being an important indicator of condition (Gaillard et al., 2000; Nussey et al., 2011), may experience strong selection pressure both in early life and late life (Jebb et al., 2021; Merilä et al., 2001; Ronget et al., 2018). In SW, juvenile body mass predicts survival till adulthood (Brown et al., 2021a), i.e., strong selection on body mass. Thus, body mass is hypothesised to experience a more rapid waning of selection pressure and ageing rate according to Moorad & Ravindran (2022). Yet, prediction two is equally plausible. We can expect immune traits to experience strong selection, as the benefit of an optimized immune function for survival and fitness is extremely apparent (McKean & Lazzaro, 2011; Seppälä, 2015; Sheldon & Verhulst, 1996).

Nevertheless, the strength of selection pressure on traits in specific populations can be difficult to predict. Although most studies suggest that body mass is under strong selection, there is also evidence suggesting low association between fitness and body mass (Boratyński & Koteja, 2010; Cox & Calsbeek, 2015; Warkentin et al., 2016). Likewise, the strength of selection on immune traits may depend on the environment and the strength of the immune challenge (Langeloh et al., 2023; Roast et al., 2020; Seppälä, 2015). In the SW, there was no evidence that avian malaria presence affected adult warbler survival (Hammers et al., 2016), which may indicate low strength of immune challenge, hence low covariation between immunity and fitness in warblers. Widely speaking, selection on a trait can be dependent on population dynamics and environment (Wright et al., 2019), making it difficult to draw broader conclusions. We currently lack understanding of trait-specific selection to definitively predict how selection determines the rate and onset of senescence or ageing patterns across traits. Nonetheless, we can predict that the trait with similar ageing trajectories may share age-specific selection pressure patterns and vice versa.

### Caveats and future directions

Senescence refers to a decline in a function in an organism that results in a decline in survival and reproductive success (Ricklefs, 2008). Therefore, inference of trait senescence is limited in our present study as for four out of the seven traits that showed ageing, we have no evidence of a causative link between trait ageing and survival/reproduction decreases. While we attempted to control for selective disappearance to the best of our ability, the age-by-lifespan interaction models were not compatible at the time of writing with the downstream analyses. Thus, our results may not reflect true ageing trajectories perfectly, so our asynchrony of ageing results should be interpreted with caution. Additionally, we also are not able to sample all traits and ages equally in a wild system, which may affect estimation of age trajectories. To understand the impact of our gaps in sampling, we ran a sensitivity analysis with a reduced dataset of the same individuals across all traits. We found that the ageing trajectories remained largely the same with the exception of survival. Thus the conclusions of our study did not change (Figure S7, Tables S39–S49). While a lab environment may provide opportunities to sample all traits equally and reduce noise in data from environmental variation, ageing mechanisms from standard laboratory environments often do not extend to wild conditions (Briga & Verhulst, 2015). Therefore, studies in wild populations are still essential to understanding evolution of ageing.

Further studies are needed to test how age-specific selection pressure on traits explains the asynchrony of ageing (Moorad & Ravindran, 2022). Additionally, genetic correlations within and between age classes, differential energy allocation and trade-offs may affect the evolution of asynchrony of senescence (Charlesworth, 2001; Hayward et al., 2015; Moorad & Ravindran, 2022). Testing for presence of genetic correlations between traits may elucidate the genetic architecture of asynchrony of senescence. Understanding potential nonevolutionary causes of asynchrony of senescence is also instrumental in providing insight to asynchronous ageing (Moorad & Ravindran, 2022). For example, since telomere senescence rates in the SW are affected by the environment (Brown et al., 2022), this study could be extended to test the environmental effects on senescence rates across various traits to examine whether environmental damage leads to differences in trait ageing. Additionally, a path analysis or structural equation modeling how individual trait ageing contributes to the timing of actuarial senescence would provide insight on the mechanistic explanation of individual senescence (Lemaître & Gaillard, 2017).

## Supplementary Material

qrag029_Supplemental_File

## Data Availability

The data and code underlying this article are available on the following repositories: GitHub: https://github.com/Clairetls/Asynchrony-of-ageing-code.git and Zenodo https://zenodo.org/records/21101620
