# Supplementary material for: Asynchrony of ageing among traits in a wild bird population
Source: Evol Lett. 2026 Jul 21;10(5):506–20. doi: 10.1093/evlett/qrag029 (PMC13629235; doi:10.1093/evlett/qrag029)
Supplement: Supplementary file 1 [file qrag029_supplemental_file.docx]

Supplementary material

Figure S1. Mean lifespan of Seychelles warblers calculated from cumulative datasets from 1997- 2022. This graph includes individuals that survived past 6 months of age. The dots indicate the mean lifespan of the dataset and error bars denote the standard error.

Figure S2 Distribution of lifespan of Seychelles warblers across the cohorts of 1981-2022.

Figure S3. Heatmap of pairwise correlations between traits with repeated measures in the Seychelles warbler. The colour and gradient denote the direction and strength of the correlation, respectively. Red stands for negative correlations while blue for positive correlations.

Figure S4. Distribution of sample size across ages for all traits. Overall sample size and number of unique individuals can be found in table S1. No individuals were caught after 17 years, but re-sighting rates and parentage analyses allow survival and ARS (annual reproductive success) data to be calculated at older ages.

Figure S5 Model curve plots showing sex-specific ageing trajectories across all traits.

Figure S6. Derivatives of the GAM models showing sex specific ageing trajectory.

Figure S7. Ageing trajectories from the sensitivity analysis using reduced datasets containing the same individuals across all traits.

Table S1. Table showing sample size and number of unique individuals for each trait.

| Trait | Sample size | Number of unique individuals |
| --- | --- | --- |
| Body mass | 2559 | 1551 |
| Wing length | 2622 | 1560 |
| Right tarsus | 2639 | 1299 |
| Fat score | 1453 | 705 |
| Oxidative stress (ROMs) | 639 | 375 |
| Antioxidant capacity (OXYs) | 638 | 375 |
| Haematocrit | 1744 | 916 |
| Buffy coat | 1744 | 916 |
| Malaria presence | 3179 | 1784 |
| Telomere length | 1762 | 1056 |
| Provisioning rate | 1004 | 702 |
| Annual reproductive success (ARS) | 5176 | 721 |
| Annual survival | 5175 | 3334 |

For tables S2-14, Significance of terms is indicated by the asterisks (*) and p values (<0.05). Significance of smooth terms indicates whether the slope deviates significantly from a linear effect. Significance of parametric coefficients indicates the significance of the control variable. N at the bottom of the table indicates the sample size of the dataset used in the model. Sex Estimate 1 indicates male and 0 indicates female.

Table S2 GAM model summary of body mass.

Table S3. GAM model summary of wing length.

Table S4. GAM model summary of right tarsus length

Table S5. Gam model summary for fat score

Table S6. GAM model summary of ROMs (oxidative species).

Table S7. GAM model summary of OXYs (antioxidant capacity).

Table S8. GAM model summary of haematocrit.

Table S9. GAM model summary of buffy coat.

Table S10. GAM model summary for malaria.

Table S11. GAM model summary for telomere length. s(Whodunnit) indicates the random effect for technician identity.

Table S12. GAM model summary for provisioning rate. The term nr_helpers is for the number of helpers in the breed group.

Table S13. GAM model summary for annual reproductive success. nr fledglings indicates the number of fledglings produced that year, a control for yearly quality. AFR stands for age at first reproduction and ALR is age at last reproduction.

Table S14. GAM model summary for survival.

Table S15. Summary table of bivariate GAM between body mass and provisioning rate. Terms with .1 are the model estimates for provisioning rate while the terms without .1 are model estimates for body mass.

Table S16 Body mass model S

Table S17 Wing length model S. Model with sex interaction term

Table S18 Tarsus model S

Table S19 Fat model S

Table s20 ROMs model S

Table S21 Oxys model S

Table S22 Buffy coat model S

Table S23 Malaria model S

Table S24 RTL model S

Table S25 Provisioning model S

Table S26 Survival model S

Table S27 Body mass GAM with age and lifespan interaction.

Table S28 Wing length GAM including age and lifespan interaction.

Table S29 Tarsus length GAM including age and lifespan interaction.

Table S30 Fat score GAM including age and lifespan interaction.

Table S31 GAM model summary of ROM including age and lifespan interaction

Table S32 GAM model summary of OXYs including age and lifespan interaction

Table S33 GAM model summary of Haematocrit including age and lifespan interaction

Table S34 GAM model summary of Buffy coat including age and lifespan interaction

Table S35 GAM model summary of Malaria including age and lifespan interaction

Table S36 GAM model summary of RTL including age and lifespan interaction

Table S37 GAM model summary of Provisioning rate including age and lifespan interaction

Table S38 GAM model summary of Annual reproductive success including an interaction term between age and age of first and last reproduction

Table S39 GAM model table for sensitivity analysis on body mass ageing trajectory using a reduced dataset

Table S40 GAM model table for sensitivity analysis on wing length ageing trajectory using a reduced dataset

Table S41 GAM model table for sensitivity analysis on tarsus length ageing trajectory using a reduced dataset

Table S42 GAM model table for sensitivity analysis on fat score ageing trajectory using a reduced dataset

Table S43 GAM model table for sensitivity analysis on haematocrit ageing trajectory using a reduced dataset

Table S44 GAM model table for sensitivity analysis on buffy coat ageing trajectory using a reduced dataset

Table S45 GAM model table for sensitivity analysis on malaria ageing trajectory using a reduced dataset

Table S46 GAM model table for sensitivity analysis on relative telomere length ageing trajectory using a reduced dataset

Table S47 GAM model table for sensitivity analysis on provisioning rate ageing trajectory using a reduced dataset

Table S48 GAM model table for sensitivity analysis on annual reproductive success ageing trajectory using a reduced dataset

Table S49 GAM model table for sensitivity analysis on survival ageing trajectory using a reduced dataset
